# The National Kidney Foundation of Illinois KidneyMobile: a mobile resource for community based screenings of chronic kidney disease and its risk factors

**DOI:** 10.1186/s12882-018-1079-y

**Published:** 2018-10-25

**Authors:** Swati Lederer, Laurie Ruggiero, Nicole M. Sisen, Nancy Lepain, Kate Grubbs O’Connor, Yamin Wang, Jinsong Chen, James P. Lash, Michael J. Fischer

**Affiliations:** 1 Center of Innovation for Complex Chronic Healthcare, Jesse Brown VA Medical Center, Chicago, IL USA; 2 Edward Hines Jr. VA Hospital, Hines, IL USA; 3 Department of Medicine, University of Illinois at Chicago, College of Medicine, Chicago, IL USA; 4 Department of Medicine, VA North Texas Healthcare System, 4500 South Lancaster Ave, MC 111G1, Dallas, TX 75216 USA; 5 National Kidney Foundation of Illinois, Chicago, IL USA; 6 Community Health Sciences Division/Institute for Health Research and Policy, School of Public Health, University of Illinois at Chicago, Chicago, IL USA; 7 Behavioral Health and Nutrition, College of Health Sciences, University of Delaware, Newark, DE USA

**Keywords:** Kidney disease, Diabetes, Hypertension, Screening, Awareness

## Abstract

**Background:**

Early detection and treatment of chronic kidney disease (CKD) and its risk factors improves outcomes; however, many high-risk individuals lack access to healthcare. The National Kidney Foundation of Illinois (NKFI) developed the KidneyMobile (KM) to conduct community-based screenings, provide disease education, and facilitate follow-up appointments for diabetes, hypertension, and CKD.

**Methods:**

Cross-sectional design. Adults > = 18 years of age participated in NKFI KM screenings across Illinois between 2005 and 2011. Sociodemographic and medical history were self-reported using structured interviews; laboratory data and blood pressure were assessed using standard procedures.

**Results:**

Among 20,770 participants, mean age was 53.5 years, 68% were female, 49% were African-American or Hispanic, 21% primarily spoke Spanish, and at least 27% lacked health insurance. Seventy-eight percent of participants with elevated blood pressure (≥ 140/90 mmHg) were aware of having hypertension, 93% of participants with abnormal blood glucose (fasting glucose > 126 mg/dl or a random glucose of > 200 mg/dL) were aware of having diabetes, and 19% of participants with albuminuria (> 30 mg/gm) were aware of having CKD. In participants reporting hypertension, 47% had blood pressure ≥ 140/90 mmHg, and in those reporting diabetes, 56% had blood glucose ≥ 130 mg/dl (fasting) or ≥ 180 mg/dl (random). Among 4937 participants with abnormal screening findings that participated in follow-up interviews, 69% reported having further medical evaluation.

**Conclusions:**

A high-risk disadvantaged population is being reached by the NKFI KidneyMobile and connected with healthcare services. A significant proportion of participants were newly informed of having abnormal results suggestive of diabetes, hypertension, and/or CKD or that their diabetes and hypertension were inadequately controlled.

## Background

Chronic kidney disease (CKD) is a common and costly disease affecting approximately 14% of the adult population in the United States and accounting for approximately 20% of Medicare Part A and B costs [1]. Despite the significant utilization of healthcare services among this patient population, CKD continues to be strongly associated with poorer patient outcomes including an increased risk of death, cardiovascular events, end-stage kidney disease (ESKD), and hospitalizations [1, 2].

Large screening initiatives such as the National Health and Nutrition Examination Survey (NHANES) and the Kidney Early Evaluation Program (KEEP) have found that most adults afflicted with CKD are unaware of their diagnosis [3–5]. Furthermore, awareness of comorbid conditions that increase risk of CKD, such as diabetes and hypertension, is also suboptimal. The under-recognition and delayed treatment of CKD and its associated health problems hastens disease progression and contributes to the growth of the ESKD population [3, 5–10]. Studies also show that low socio-economic subgroups, African Americans, and Hispanics have lower disease awareness and carry a higher burden of ESKD compared to the general population [11–13]. These vulnerable populations may have barriers to accessing healthcare resulting in later diagnoses of medical conditions [11–14]. Improving awareness of CKD and its comorbid conditions allows patients to seek the appropriate medical management, obtain fundamental disease knowledge, and participate in disease management.

Mobile screening initiatives have been successful in identifying individuals who may not otherwise seek medical care and facilitating the early diagnosis of silent diseases (e.g., CKD, hypertension, diabetes, and breast cancer) [15–19]. While findings from broad screening initiatives for hypertension, diabetes, and kidney disease are well-known, community-based mobile screening programs targeting under-insured individuals without access to primary care providers have not been well characterized. In addition to facilitating early diagnoses, these initiatives often provide individuals with health education and primary care follow up. Community-based screening initiatives also help educate public health officials and health providers about the burden of disease within specific communities and may inform the development and implementation of targeted interventions.

The National Kidney Foundation of Illinois (NKFI) developed a KidneyMobile (KM) as a mobile screening vehicle in 2005 to enhance the detection of hypertension, diabetes, and kidney disease in high-risk and vulnerable communities across Illinois. Working with community partners, the NKFI KM has conducted almost 41,000 screenings for diabetes, hypertension, and kidney disease in underserved areas between the years of 2005–2014. The KM also provides interactive educational activities and facilitates healthcare provider follow up for participants with abnormal screening results who lack access to healthcare services. To characterize the population reached by screenings and to evaluate the impact of this program, we examined data collected from 20,770 participating individuals between the years of 2005–2011.

## Methods

### Study sample and design

We conducted a cross-sectional analysis of data from NKFI KM participants throughout Illinois between 2005 and 2011. A total of 23,166 adult participants over the age of 18 were voluntarily screened. The NKFI KM was developed to conduct free screenings, provide disease education (diabetes, hypertension, CKD), and facilitate healthcare appointments for participants. The NKFI partnered with federally qualified health centers, health departments, and community hospitals in underserved areas to ensure that we targeted a high-risk population, characterized by substantial proportions of underinsured as well as uninsured adults without ready access to health care. Examples of such high-risk groups in our screenings included urban communities with significant percentages of African American and Hispanic adults subject to health disparities and rural communities with geographic barriers to health care services. Additionally, screening sites were selected based on their ability to provide resources for the screening day (e.g., volunteers, space, equipment) and post-screening follow up care.

A screening visit consisted of three stages and was led by a nurse who was assisted by trained healthcare volunteers. First, sociodemographic information, medical history, vital signs, anthropometric measures, and laboratory tests were obtained from participants. Second, educational information related to healthy living, hypertension, diabetes, and CKD was provided to participants. Third, participants with abnormal screening results met individually with a healthcare provider for further consultation. Those participants who had health insurance were directed back to their primary care provider, while those participants without health insurance were provided with a healthcare referral for a follow up appointment at a federally qualified health center or community hospital. Finally, attempts were made to reach participants with abnormal screening tests, by telephone for follow up to ensure that they had visited a healthcare provider following screening.

IRB approval from the University of Illinois at Chicago was obtained for analysis of these screening data, which had originally been obtained for non-research purposes.

### Variables and data sources

Sociodemographic characteristics were self-reported by participants on a 19-item questionnaire at the initial screening visit. These characteristics included age, sex, race/ethnicity (white, African-American, Hispanic, Asian/Pacific Islander, other), primary language (English, Spanish, other), presence of health insurance and primary provider, and personal or family history of hypertension, diabetes, kidney disease. Height, weight, and manual blood pressure were measured by a trained nurse per standard protocols [20]. If the systolic blood pressure was greater than 160 mmHg, the reading was repeated. Blood glucose measurements were drawn and analyzed via the One Touch Ultra-2 device [21]. After collecting a clean catch midstream urine sample from all participants, a urine dipstick and a urine microalbumin were analyzed onsite by Clinitek device [22]. Any participant with albuminuria (defined below), personal history of diabetes, or an abnormal serum glucose testing was offered a blood test to measure serum creatinine, either on site by the Abbott I-stat Chem 8+ or by a nearby hospital laboratory [23].

### Definition of variables

We defined prevalent hypertension as participants who self-reported a history of hypertension, or those who had a systolic blood pressure ≥ 140 mmHg or a diastolic pressure ≥ 90 mmHg, while recognizing that a definitive diagnosis of hypertension cannot be made by a single blood pressure measurement. Among those participants with prevalent hypertension, awareness of hypertension was defined as an affirmative to the following questionnaire item: “Have you ever been told you have high blood pressure or hypertension?”. Among participants who reported a history of hypertension, control of the disease was defined as having both a systolic blood pressure of < 140 mmHg and a diastolic blood pressure of < 90 mmHg.

We defined prevalent diabetes as participants who self-reported a history of diabetes, a fasting serum glucose ≥ 126 mg/dl or a non-fasting glucose ≥ 200 mg/dl, recognizing that a definitive diagnosis of diabetes cannot be made by a single blood glucose measurement and requires confirmatory testing [24]. Among participants with prevalent diabetes, awareness of diabetes was defined as an affirmative to the following questionnaire item: “Have you ever been told that you have diabetes or high blood sugar?”. Among those participants reporting a history of diabetes, control of the disease was defined as a fasting blood glucose of < 130 mg/dl or a non-fasting blood glucose of < 180 mg/dl [24].

We defined prevalent chronic kidney disease as participants self-reporting a history of kidney disease or those with a urine albumin to creatinine ratio of ≥ 30 mg/gm, recognizing that diagnosis of kidney disease cannot be made by a single urine measurement [25]. Among those with prevalent CKD, awareness of the condition was defined as an affirmative to the following questionnaire item: “Have you ever been told you have kidney disease?”

A small subset of the total cohort underwent blood testing to measure serum creatinine. Among those participants, eGFR was calculated using the modification of diet in renal disease (MDRD) equation [26].

### Statistical analysis

All sociodemographic, clinical and laboratory data from participants screened was organized into analytic datasets. Participant characteristics were summarized using means with standard error for continuous variables and frequency distribution with percentages for categorical variables. Characteristics were compared across strata of albuminuria by Chi-Squared or ANOVA testing as appropriate. Missing values occurred under the following circumstances: i) when a participant failed to answer a question on a reporting form, ii) when a physical measure was not obtained, iii) when a laboratory test was not performed. All statistical summaries were conducted using SAS, version 9.1 (Cary, NC, USA).

## Results

### Participant sociodemographic and clinical characteristics

**Fig. 1:**
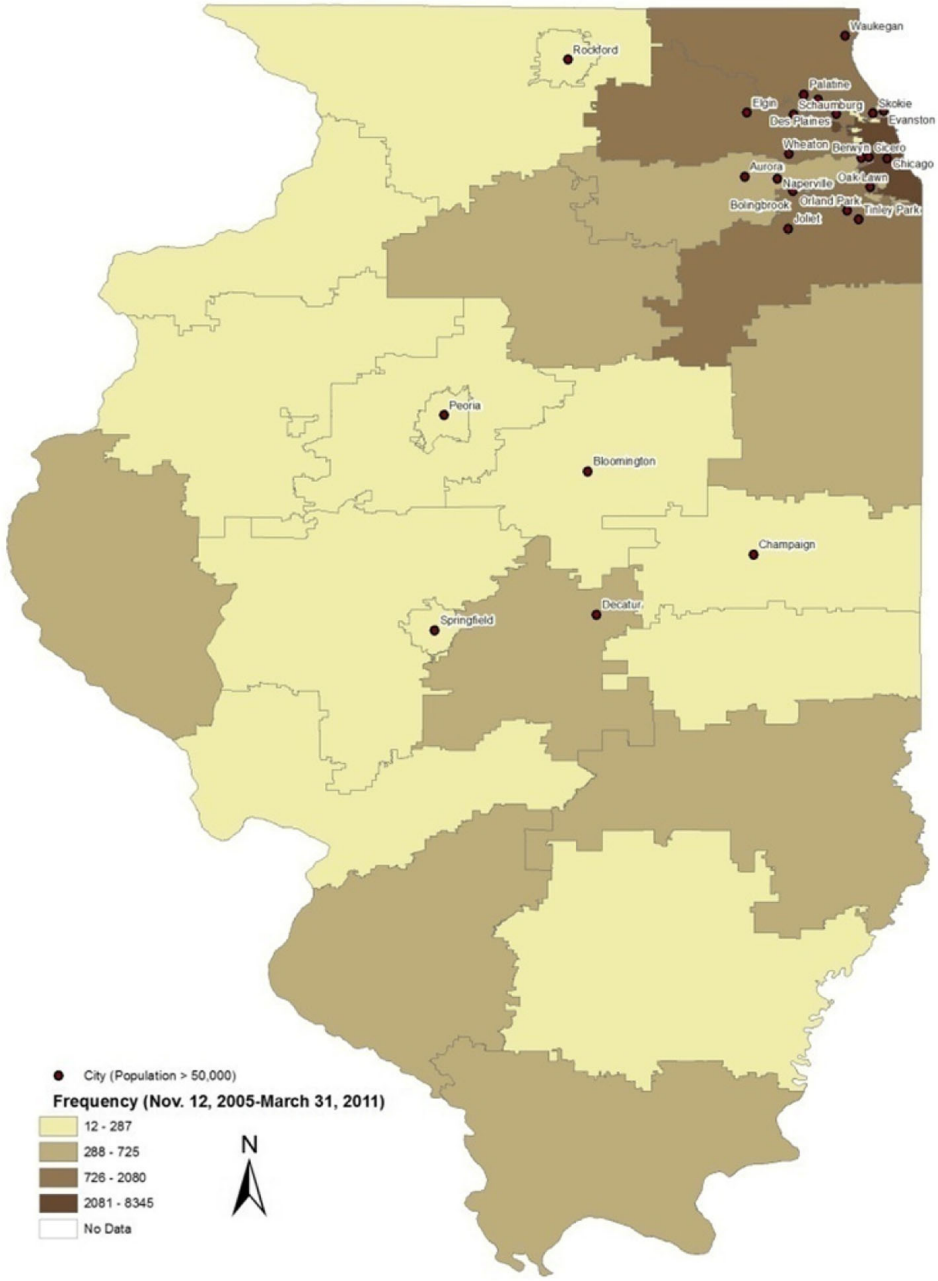
Distribution of KM screenings across Illinois

Out of a total of 23,166 adults participating in KidneyMobile screenings across Illinois from 2005 to 2011, 20,770 had complete data regarding age, sex, and race/ethnicity, and were included in the final analytic cohort. Figure 1 illustrates the geographic distribution of the KM screening across Illinois. Nearly half of the participants (54%) were from the greater Chicago area, 44% were from the remainder of Illinois, and 2% were not known.

The mean age of participants was 53.5 years, approximately 68% were female, 58% were of non-White racial/ethnic background, and 21% reported Spanish as their primary language (Table 1). A large percentage of participants either lacked health insurance (27%) or were unsure if they had active insurance (13%), and 50% did not have or did not know if they had a primary care provider. While 39% of participants reported a history of hypertension, 20% and only 5% of participants reported a history of diabetes mellitus and kidney disease, respectively. A family history of hypertension (54%) and diabetes (43%) were common among participants, but a family history of kidney disease was reported by only 12%. Among all participants, 31% were overweight (BMI 25–29.9), 29% were obese (BMI 30–39.9), and 6% were morbidly obese (BMI > 40). The mean systolic and diastolic blood pressure among participants was 127.5 mmHg (se 0.13) and 76.6 mmHg (se 0.08), respectively. Most participants (72%) had fasting or non-fasting blood glucose levels within normal range.

**Table 1:** Participant Sociodemographic and Clinical Characteristics Stratified by Albuminuria

Characteristic	ALL N (%) or Mean (se)					Unknown/Not tested N (%) or Mean (s.e.)
		Urine albumin/crt < 30 N (%)	Urine albumin/crt 30–300 N (%)	Urine albumin/crt > 300 N (%)	*p*-value	
Total	20,770	15,396 (74.1)	4173 (20.1)	311 (1.5)		890 (4.3)
Age^a^	53.5 (0.12)	53.09 (0.13)	55.26 (0.27)	61.42 (0.94)	< 0.001	50.35 (0.58)
Sex						
Male	6717 (32.3)	5051 (32.8)	1246 (29.9)	120 (38.6)	< 0.001	300 (33.7)
Female	14,053 (67.7)	10,345 (67.2)	2927 (70.1)	191 (61.4)		590 (66.3)
Race/ethnicity						
White (non-Hispanic)	8801 (42.4)	6673 (43.3)	1693 (40.6)	144 (46.3)	< 0.001	291 (32.7)
African-American	4346 (20.9)	3062 (19.9)	1015 (24.3)	55 (17.7)		214 (24.04)
Hispanic	5838 (28.1)	4365 (28.4)	1130 (27.1)	78 (25.1)		265 (29.8)
Asian/Pacific Islander	934 (4.5)	713 (4.63)	156 (3.8)	20 (6.4)		45 (5.1)
Other/unknown	505 (2.43)	350 (2.3)	98 (2.4)	5 (1.6)		52 (5.8)
Primary language						
English	13,736 (66.1)	10,131 (65.8)	2944 (70.6)	222 (71.4)	< 0.001	439 (49.3)
Spanish	4417 (21.3)	3289 (21.36)	890 (21.3)	64 (20.6)		174 (19.6)
Other/unknown	2108 (10.15)	1580 (10.26)	250 (6.0)	11 (3.5)		267 (30.0)
Health insurance						
Yes	12,524 (60.3)	9399 (61.1)	2583 (61.9)	184 (59.2)	0.1130	358 (40.2)
No	5561 (26.8)	4171 (27.1)	1156 (27.7)	90 (28.9)		144 (16.2)
Unknown	2685 (12.9)	1826 (11.9)	434 (10.4)	37 (11.9)		388 (43.6)
Primary Provider						
Yes	10,311 (49.6)	7722 (50.2)	2118 (50.8)	164 (52.7)	< 0.001	307 (34.5)
No	3936 (19.0)	3027 (19.7)	733 (17.6)	38 (12.2)		138 (15.5)
Unknown	6523 (31.4)	4647 (30.2)	1322 (31.7)	109 (35.1)		445 (50.0)
Hypertension						
Yes	8053 (38.8)	5596 (36.4)	1968 (47.2)	204 (65.6)	< 0.001	285 (32.0)
No	11,742 (56.5)	9122 (59.3)	2048 (49.1)	95 (30.6)		477 (53.6)
Unknown	975 (4.7)	678 (4.4)	157 (3.8)	12 (3.9)		128 (14.4)
Diabetes						
Yes	4187 (20.2)	2752 (17.9)	1149 (27.5)	155 (49.8)	< 0.001	32 (3.6)
No	15,577 (75.0)	11,972 (77.8)	2853 (68.4)	145 (46.6)		524 (58.9)
Unknown	1006 (4.8)	672 (4.4)	171 (4.1)	11 (3.5)		334 (37.5)
Kidney disease						
Yes	990 (4.8)	625 (4.1)	272 (6.5)	61 (19.6)	< 0.001	32 (3.6)
No	18,065 (87.0)	13,696 (89.0)	3620 (86.8)	225 (72.4)		524 (58.9)
Unknown	1715 (8.2)	1075 (7.0)	281 (6.7)	25 (8.0)		334 (37.5)
Family history of hypertension						
Yes	11,183 (53.8)	8258 (53.6)	2358 (56.6)	181 (58.0)	< 0.001	386 (43.4)
No	6836 (32.9)	5153 (33.5)	1312 (31.4)	78 (25.1)		293 (32.9)
Unknown	2751 (13.2)	1985 (12.9)	503 (12.1)	52 (16.7)		211 (23.7)
Family history of diabetes						
Yes	8837 (42.5)	6466 (42.0)	1956 (46.9)	164 (52.7)	< 0.001	251 (28.2)
No	8116 (39.1)	6158 (40.0)	1620 (38.8)	97 (31.2)		241 (27.1)
Unknown	3817 (18.4)	2771 (18.0)	597 (14.3)	50 (16.7)		398 (44.7)
Family history of kidney disease, transplant, or dialysis						
Yes	2477 (11.9)	1846 (12.0)	507 (12.2)	59 (19.0)	< 0.001	65 (7.3)
No	13,070 (62.9)	9763 (63.4)	2757 (66.1)	179 (57.6)		371 (41.7)
Unknown	5223 (25.2)	3787 (24.6)	909 (21.8)	73 (23.5)		454 (51.0)
BMI^a^	29.4 (0.05)	29.2 (0.05)	30.1 (0.11)	29.6 (0.4)	< 0.001	29.1 (0.29)
BMI Category						
< 25	4865 (23.4)	3701 (24.0)	926 (22.2)	74 (23.8)	< 0.001	164 (18.4)
25–29.9	6470 (31.2)	5007 (32.5)	1189 (28.5)	88 (28.3)		186 (20.9)
30–39.9	6024 (29.0)	4447 (28.9)	1332 (31.9)	86 (27.7)		159 (17.9)
> =40	1273 (6.1)	871 (5.7)	342 (8.2)	24 (7.7)		36 (4.0)
Unknown	2138 (10.3)	1370 (8.9)	384 (9.2)	39 (12.5)		345 (38.8)
Systolic blood pressure, mm Hg^1^	127.5 (0.13)	126.3 (0.14)	130.8 (0.31)	143.3 (1.33)	< 0.001	126.2 (0.83)
Systolic blood pressure categories, mmHg						
< 120	6186 (29.8)	4868 (31.6)	1066 (25.6)	37 (11.9)	< 0.001	215 (24.2)
120–139	8513 (41.0)	6489 (42.2)	1706 (40.9)	99 (31.8)		219 (24.6)
140–159	3662 (17.6)	2609 (17.0)	866 (20.8)	86 (27.7)		101 (11.4)
160–179	952 (4.6)	591 (3.8)	290 (7.0)	49 (15.8)		22 (2.5)
> = 180	237 (1.1)	106 (0.7)	92 (2.2)	25 (8.0)		14 (1.6)
Unknown	1220 (5.9)	733 (4.8)	153 (3.7)	15 (4.8)		319 (35.8)
Diastolic blood pressure mm Hg^a^	76.6 (0.08)	76.1 (0.09)	77.8 (0.19)	80.4 (0.74)	< 0.001	77.6 (0.5)
Diastolic blood pressure categories						
< 80	11,022 (53.1)	8468 (55.0)	2117 (50.7)	130 (41.8)	< 0.001	307 (34.5)
80–89	6023 (29.0)	4530 (29.4)	1220 (29.2)	91 (29.3)		182 (20.5)
90–99	2003 (9.6)	1417 (9.2)	480 (11.5)	47 (15.1)		59 (6.6)
> =100	575 (2.8)	317 (2.1)	203 (4.9)	26 (8.4)		29 (3.3)
Unknown	1147 (5.5)	664 (4.3)	153 (3.7)	17 (5.5)		313 (35.2)
Blood glucose level, gm/dL^b^						
F < 100 or NF < 140	15,013 (72.3)	11,596 (75.3)	2795 (67.0)	168 (54.0)	< 0.001	307 (34.5)
F = 100–126 or NF = 140–200	2488 (12.0)	1771 (11.5)	600 (14.4)	46 (14.8)		182 (20.5)
F > 126 or NF > 200	1138 (5.5)	607 (3.9)	432 (10.4)	68 (21.9)		59 (6.6)
Unknown	2123 (10.2)	1417 (9.2)	343 (8.2)	29 (9.3)		313 (35.2)

^a^mean (standard error)

^b^F = fasting, NF = non-fasting

Most participants (74%) had no evidence of albuminuria, whereas 20% were found to have between 30 and 300 mg/gm of albuminuria and 1.5% were found to have > 300 mg/gm of albuminuria. Approximately 4% of screening participants did not have a urine microalbumin screen. Participant characteristics differed substantially across strata of albuminuria (Table 1). Mean age was significantly higher with increasing albuminuria (*p* < 0.001). Participants with albuminuria (> 30 mg/gm) were more likely to report a personal history of hypertension, diabetes, and kidney disease compared to participants without albuminuria (*p* < 0.001). Albuminuria was also more common among participants who reported a family history of hypertension, diabetes, and kidney disease. Less consistent changes were observed with other sociodemographic characteristics.

Mean systolic and diastolic blood pressure increased significantly with higher albuminuria. The mean systolic blood pressure for participants without albuminuria was 126.3 mmHg, 130.8 mmHg for those with 30-300 mg/gm albuminuria, and 143.3 mmHg for those with > 300 mg/gm albuminuria (*p*-value < 0.001). Diastolic blood pressure also increased with higher albuminuria: 76.1 mmHg in participants without albuminuria, 77.8 mmHg among those with 30-300 mg/gm of albuminuria, and 80.4 mmHg among those with > 300 mg/gm of albuminuria (*p*-value < 0.001).

Increasing albuminuria was associated with poorer blood glucose control. Four percent of participants without albuminuria had a fasting blood glucose of > 126 mg/dl or a non-fasting glucose > 200, whereas 10% of participants with 30-300 mg/gm and 22% of those with > 300 mg/gm of albuminuria had abnormal blood glucose levels.

### Estimated glomerular filtration rate among a subgroup of patients with abnormal screening results

A total of 4014 participants with albuminuria and either hypertension or diabetes underwent serum blood testing and calculation of eGFR (Table 2). There was a significant association between albuminuria and eGFR (*p* < 0.001). Among participants without albuminuria, most (85%) had an eGFR > = 60 ml/min/1.73m^2^, whereas 14% had an eGFR between 30 and 59 ml/min/1.73m^2^ and 1% had an eGFR < 30 ml/min/1.73m^2^. Twenty-six percent of participants with 30-300 mg/gm of albuminuria and 40 % of those with > 300 mg/gm of albuminuria had an eGFR between 30 and 59 ml/min/1.73m^2^, and almost 20 % of participants with > 300 mg/gm of albuminuria had an eGFR of < 30 ml/min/1.73m^2^.

**Table 2:** Estimated GFR among a subgroup of 4014 participants with abnormal lab results

	ALL N (%) or Mean (se)	Urine albumin/crt < 30 N (%)	Urine albumin/crt 30–300 N (%)	Urine albumin/crt > 300 N (%)	*p*-value	Unknown/Not tested N (%) or Mean (s.e.)
Total	4014	2873	982	311		70
eGFR, ml/min/1.73m^2^						
> = 60	3203 (79.8)	2427 (84.5)	692 (70.5)	36 (40.4)	< 0.001	48
30–59	723 (18.0)	414 (14.4)	252 (25.7)	36 (40.4)		21
< = 29	88 (2.2)	32 (1.1)	38 (3.9)	17 (19.1)		1

### Prevalence, awareness, and control of diabetes, hypertension, and kidney disease among participants

**Fig. 2:**
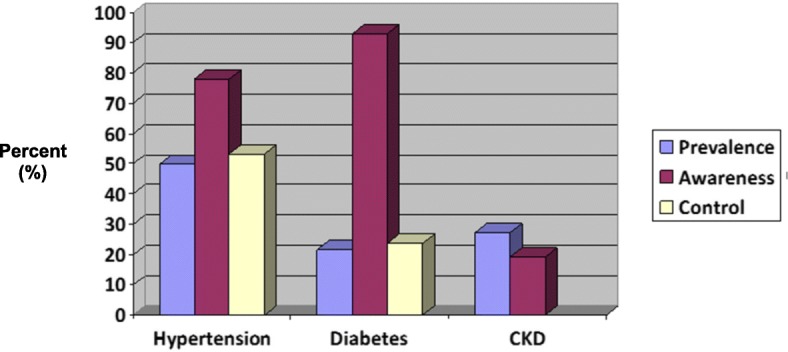
Prevalence, awareness, and control of hypertension, diabetes, and chronic kidney disease

Fifty percent of participants had prevalent hypertension, as defined by self-reported history of hypertension, systolic blood pressure ≥ 140 mmHg, or a diastolic blood pressure ≥ 90 mmHg. Of those with hypertension, 78% were aware of a hypertension diagnosis prior to the screening. Among patients with a reported history of hypertension, about half (53%) were well-controlled with a systolic blood pressure of < 140 mmHg and a diastolic blood pressure of < 90 mmHg (Fig. 2).

Twenty-two percent of study participants had prevalent diabetes, as defined by a self-reported history of diabetes, a fasting blood glucose > 126 mg/dl, or a non-fasting blood glucose of > 200 mg/dl. Among those with diabetes, 93% reported awareness of diabetes diagnosis prior to the screening. Only 24% of participants who reported diabetes were well-controlled with a fasting BS < 130 or a non-fasting BS < 180 (Fig. 2).

Twenty-seven percent of participants had prevalent CKD, as defined by a reported history of CKD or a urine albumin/creatinine ratio of > 30 mg/gm. Only 19% of participants with CKD were aware of this diagnosis prior to the screening. (Fig. 2).

### Access to healthcare among participants with prevalent hypertension, diabetes, and CKD

**Fig. 3:**
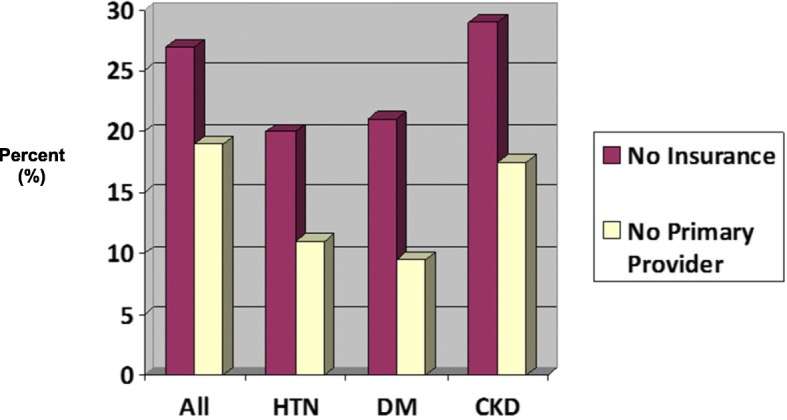
Lack of health care access among participants with prevalent hypertension, diabetes, and chronic kidney disease

Compared to all participants, access to healthcare was better among participants with hypertension and diabetes and similar among participants with CKD. Approximately 19% of participants with prevalent hypertension, 21% of those with diabetes, and 29% with CKD reported lacking health insurance, whereas approximately 27% of all participants reported not having health insurance. Eleven percent of participants with hypertension, 10% with diabetes, and 18% with CKD, reported not having a primary care provider, while 19% of all participants reported not having one (Fig. 3).

### Post-screening telephone follow up

A total of 4937 participants with abnormal screening findings (e.g., elevated blood pressure, elevated blood glucose readings, and abnormal urine findings) were reached by telephone for a follow up interview after their screening. Of these participants, 3387 or 68.6% reported follow up with a healthcare provider, whereas 1138 participants (23.1%) reported no follow up. Of the participants who obtained healthcare post-screening, 1580 (46.6%) had repeat testing and 1317 (38.9%) reported initiation of treatment for their condition.

## Discussion

Among a diverse cohort of 20,770 adults across the state of Illinois who participated in health screenings by the NKFI KM, we found a significant prevalence of undetected and poorly controlled hypertension, diabetes, and kidney disease. At KM screenings, 78% of adults with prevalent hypertension were aware of their hypertension, 93% of participants with prevalent diabetes were aware of their diabetes, but only 19% of participants with prevalent CKD were aware of having the condition. Among those participants reporting hypertension, 53% had adequate blood pressure control as defined by ≤ 140/90 mmHg, and in those reporting diabetes, 24% had adequate control, as defined by a fasting blood glucose ≤ 130 mg/dl or a non-fasting blood glucose < 180 mg/dl. The NKFI KM reached an underserved population as 27% of participants reported lacking health insurance and 19% did not have access to a healthcare provider. The NKFI KM assisted many underserved participants in addressing their healthcare needs. Among five thousand individuals with abnormal findings who participated in a post-screening interview, almost 70% had a healthcare provider appointment and further evaluation arranged through the NKFI.

Hypertension and diabetes comprise leading causes of CKD, and control of these conditions is needed to prevent CKD and related complications [1, 27, 28]. Studies have shown that adults who are provided with a diagnosis of hypertension and diabetes have better control of these conditions [16], which in turn reduces the incidence of CKD and slower progression of established CKD [27, 29–31]. Similarly, individuals who are made aware of a diagnosis of kidney disease are more likely to obtain CKD care, which may help reduce their incidence of ESKD and CKD related complications [6, 32]. The positive impact of disease awareness on clinical outcomes underscores the importance of timely diagnosis of these conditions. However, it is important to note that there can be unintended results of screening, including unnecessary worry by participants, barriers to securing disability insurance for participants, and generation of additional testing and/or treatments that may not yield better outcomes. While large health screenings for diabetes, hypertension, and kidney disease such as the KEEP study are common, there are fewer examples of mobile health screenings such as the NKFI KM. Mobile health screenings offer specific advantages of being able to reach individuals without health insurance or a primary provider and who have less geographic mobility. In addition, mobile health screenings can target certain geographic areas overrepresented by disadvantaged and high-risk groups [16, 33, 34]. Our mobile screening initiative allowed us to reach a more vulnerable and disadvantaged population (e.g., lack primary provider, lack health insurance, racial/ethnic minority, non-English speaking) than that represented in broader screening initiatives such as NHANES and KEEP [35]. Furthermore, approximately 22% of KM participants were found to have albuminuria, which is markedly higher than that identified by KEEP (12%) or NHANES (10%) [35, 36].

We found a significant burden of undiagnosed hypertension, diabetes, and kidney disease among NKFI KM participants, consistent with findings from KEEP [37]. The KEEP initiative screened adult patients between the ages of 18–65 years, with either a personal or first-degree family history of diabetes, hypertension and/or chronic kidney disease across the United States. In contrast, the NKFI KidneyMobile screened all adult participants over the age of 18 irrespective of their personal or family health history throughout the state of Illinois. Among participants with abnormal screening results, KEEP investigators found that 35% of those with elevated blood pressure (> = 140/90), 2% with abnormal serum glucose (> = 180), 96% with albuminuria were unaware of a personal history of hypertension, diabetes, and/or kidney disease, respectively [37]. Using a more inclusive screening protocol, we found that 22% of participants with elevated blood pressure (> = 140/90), 7% of participants with elevated blood glucose (Fasting ≥ 126 mg/dl, Non-Fasting ≥ 200 mg/dl), and 81% of participants with albuminuria (> 30 mg/gm) were unaware of a personal history of hypertension, diabetes, and kidney disease, respectively.

It is well known that poor control of hypertension and diabetes leads to an increased risk of cardiovascular disease, ESKD, and death [27, 28]. However, control of hypertension and diabetes remains suboptimal per recent reports in representative American samples [38, 39]. Similar to our findings, other screening studies have also found suboptimal control of hypertension and diabetes among adults who have known disease. In KEEP, 64% of participants with known hypertension had elevated blood pressure (> = 140/90) and 35% of those with known diabetes had an elevated serum glucose level (glucose level ≥ 180 mg/dl) [37]. We found that 47% of participants with known hypertension had an elevated blood pressure (> = 140/90) and 76% of participants with known diabetes had inadequate control of blood glucose level (fasting > = 130 mg/dl, non-fasting > = 180 mg/dl).

While screening studies have assessed the prevalence of undetected and uncontrolled hypertension, diabetes, and kidney disease within high-risk communities [17, 40], few have coupled screening with disease education or examined the impact of screenings on participants likelihood to follow-up for further care [37, 40], All KM screenings involved interactive, nurse-led educational sessions pertaining to the prevention, detection, and management of silent chronic diseases (e.g., CKD, hypertension, diabetes, obesity, hyperlipidemia). Furthermore, as part of the NKFI KM mission, all participants were given the opportunity to obtain an on-site consultation with a healthcare professional. Individuals with abnormal anthropomorphic parameters or laboratory data also received assistance with obtaining follow up medical evaluation and treatment post-screening. Based on post-screening phone calls by the NKFI staff, these efforts appeared to be quite successful as many participants reached by phone (77%) reported having had an appointment with a healthcare provider after the NKFI KM screening, and of those, 47% reported having undergone repeat testing and 39% reported initiation of medical treatment. Although the impact of this follow-up care cannot be assessed by our study, it clearly provides an opportunity for intervening and reducing complications from these conditions. A future large study would be helpful to examine whether such screening and follow up endeavors, such as the NKFI KM, affects clinical outcomes.

While our study involved a large diverse participant sample across the state of Illinois and employed detailed data collection strategies with rigorously trained study personnel, it does have limitations. First, like other screening studies such as KEEP and NHANES, only a single anthropomorphic and laboratory measurement for diabetes, hypertension, and kidney disease were done and this does not allow for a diagnosis. Repeated measurements of blood pressure and laboratory tests are generally recommended to support a new diagnosis of hypertension, diabetes, or chronic kidney disease, which underscores the importance of the NKFI KM’s coordination of medical follow up for participants with abnormal values. Also, because HbA1C was rarely performed at screening, we relied on capillary blood glucose levels to assess presence and control of diabetes, and these values may vary greatly depending on timing of meals and anti-glycemic agent, and therefore allows for possible misclassification of diabetes prevalence and control [24]. Second, we encountered missing sociodemographic, medical history, and laboratory data that may bias our results. However, missing data occurred rarely and comprised a minority of all data. Third, serum creatinine values were only obtained in participants who had urine studies positive for albumin so we may have missed participants with non-proteinuric kidney disease and underreported the prevalence of kidney disease in this screening cohort. Also, given the mobile nature and time period of the screening initiative, measurement of serum creatinine was not calibrated or standardized in a central laboratory to IDMS, which could introduce error into reported eGFR values. Fourth, as with many screening initiatives, motivational bias may have resulted in individuals participating in more than one free screening and/or attracted individual with existing health concerns, which limits the generalizability of the results.

## Conclusion

In summary, we found a high prevalence of hypertension, diabetes, and kidney disease among participants without known disease as well as poor control of these chronic conditions in participants with known disease. Additionally, the screening and follow up procedures of the NKFI KM appear to be connecting many adults with undiagnosed and uncontrolled conditions to much needed healthcare services. These results reinforce the continued need for mobile disease screening units like the NKFI KM to reach high-risk populations who often lack regular access to healthcare. Additional studies are needed to examine the impact of such mobile screening facilities on important and well-recognized clinical outcomes over time.

## Abbreviations

CKD: Chronic Kidney Disease
eGFR: Estimated Glomerular Filtration Rate
ESKD: End-Stage Kidney Disease
KM: KidneyMobile
NKFI: National Kidney Foundation of Illinois
VA: United States Department of Veteran Affairs
